# Spatial biology in cancer epigenetics

**DOI:** 10.1002/1878-0261.70310

**Published:** 2026-07-23

**Authors:** Eva Crespo‐García, Manel Esteller

**Affiliations:** ^1^ Cancer Epigenetics Group Sant Pau Research Institute (IRSantPau) Barcelona Catalonia Spain; ^2^ Centro de Investigacion Biomedica en Red Cancer (CIBERONC) Madrid Spain; ^3^ Institucio Catalana de Recerca i Estudis Avançats (ICREA) Barcelona Catalonia Spain; ^4^ Physiological Sciences Department, School of Medicine and Health Sciences University of Barcelona (UB) Barcelona Catalonia Spain

**Keywords:** cancer, chromatin, DNA methylation, epigenetics, histone modifications, spatial biology

## Abstract

Cancer develops inside organized tissue environments wherein cellular behavior is heavily influenced by local interactions and spatially restricted regulatory programs. While bulk and single‐cell sequencing technologies have fundamentally revolutionized our understanding of tumor biology, these techniques often disrupt tissue architecture and therefore fail to capture the spatial context in which molecular processes occur. Spatial transcriptomics has provided important insights into tumor heterogeneity, microenvironmental organization, and cell‐to‐cell communication. However, gene expression alone offers only an indirect view of the regulatory mechanisms governing cellular states. The emergence of spatial epigenomic technologies now enables the investigation of chromatin accessibility, histone modifications, and DNA methylation while preserving tissue structure. Here, we discuss the current landscape of spatial epigenomics, including spatial ATAC‐seq, spatial CUT&Tag, emerging spatial CUT&RUN approaches, spatial DNA methylation profiling, and multimodal strategies integrating epigenetic, transcriptional, and proteomic information within the same tissue context. Despite remaining technical and computational challenges, continued advances are expected to establish spatial epigenomics as a powerful tool for studying cancer pathways and their regulation within intact tissues.

## Graphical abstract

Spatial epigenomics combines molecular profiling with tissue architecture to reveal how gene regulation is organized within intact tissues. In cancer, these technologies uncover the mechanisms driving tumor heterogeneity and microenvironmental interactions, opening new opportunities for biomarker discovery and precision medicine. Created in BioRender. Crespo García, E. (2026) https://BioRender.com/6l6nceh.

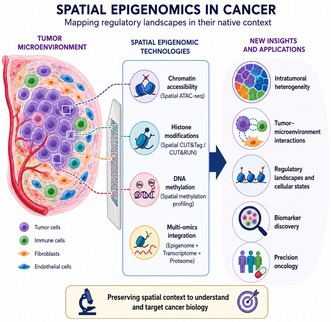

**Abbreviations**
ATAC‐seq: assay for transposase‐accessible chromatin using sequencing
CUT&RUN: Cleavage Under Targets and Release Using Nuclease
DARs: differentially accessible regions
DLBCL: diffuse large B‐cell lymphoma
eQTLs: expression quantitative trait loci
FFPE: formalin‐fixed paraffin‐embedded
GBM: Glioblastoma
ISH: *In situ* hybridization
LMD: laser microdissection
MNase: Micrococcal nuclease
mRNA: messenger RNA
spatial‐DMT: spatial DNA methylation
ST: spatial transcriptomics
TF: transcription factor
TLS: tertiary lymphoid structures

## 1 Introduction: Why spatial biology is essential to study cancer pathways

The history of cancer research dates back to ancient times, and our understanding of cancer has continuously improved as technologies to study it have advanced [1]. Major breakthroughs in cancer research have been enabled by the development of novel staining methods and improvements in microscopic observation [2]. Advances in microscopy led to the discovery of fundamental principles in tumor biology, including the confirmation that cells are the basic units of organisms, the abnormal division of cancer cells, the recognition that cancer cells originate from normal cells, and the structural differences among carcinoma subtypes [2, 3, 4].

The discovery of hematoxylin and eosin staining [5] was a major breakthrough, and it is still widely used today to look at the overall structure of tissues and how cells are organized [6]. Later on, new techniques like hybridization‐based staining and immunostaining were developed, which helped with the identification of biomarkers, detection of differences in tissues, and figuring out the best course of treatment. Over time, these techniques have been refined and are now used to inform prognosis and guide therapy. They have become essential tools in understanding the complexities of tissue architecture and cellular organization and have greatly improved our ability to diagnose and treat diseases [7, 8, 9, 10, 11, 12, 13, 14].

Beyond the visual examination of cancer tissues, cutting‐edge molecular approaches to characterize cellular composition have been developed and optimized [15]. One key advancement was the Human Genome Project generating the first complete human genome sequence which reinforced the concept that cancer arises from normal cells through the accumulation of genetic mutations and provides a reference framework to identify cancer‐associated gene alterations [16, 17]. The Cancer Genome Atlas Project, initiated in 2005 and completed in 2018, produced a comprehensive atlas of cancer‐related genomic alterations, significantly advancing the field [18].

The existence of cellular heterogeneity has been firmly established through discoveries made in the field of cancer research. This idea is further supported by the development of single‐cell sorting and sequencing technologies [19, 20, 21], which have enabled deeper insights into tumor progression [22, 23], intratumoral heterogeneity [24], and metastasis.

While many questions have been answered, the development of new technologies has simultaneously led to more unknowns about how cancer works in our bodies. Tumors were originally thought of as just a group of malignant cells, but we now know they are complex systems that can change how cells behave. This means we need to look at tumors in a different way, recognizing that they represent complex and highly organized structures that actively shape cellular mechanisms associated with cancer [25].

Growing evidence has demonstrated that the spatial context of tumor architecture plays a critical role in determining mechanisms of tumor initiation, progression, metastasis, and therapeutic response [26]. Dynamic interactions between tumor cells and their surrounding stroma establish the tumor immune microenvironment. This microenvironment exhibits diverse immune populations comprised of B cells, T cells, macrophages, and dendritic cells, which together contribute to the regulation of immune responses within tumor tissues [22, 27, 28, 29, 30, 31].

Looking at the way tissues are organized in space has helped us find special structures, like tertiary lymphoid structures (TLS), which are formed by immune cell–rich aggregates and are involved in anti‐tumor immune responses. [32]. Furthermore, the spatial organization of tumor and stromal components strongly influences the therapeutic efficacy and the development of drug resistance. This reveals the importance of spatial technologies for the discovery of predictive and therapeutic biomarkers [31, 33, 34, 35].

Importantly, clinical outcomes are closely linked to the spatial composition and organization of different cancer subtypes [36, 37, 38]. Consequently, tissue architectures are now being analyzed extensively using spatially resolved technologies. By employing them, it is possible to decode tumor microenvironmental interactions, characterize heterogeneity, and identify novel biomarkers with diagnostic and therapeutic potential.

**Fig. 1:**
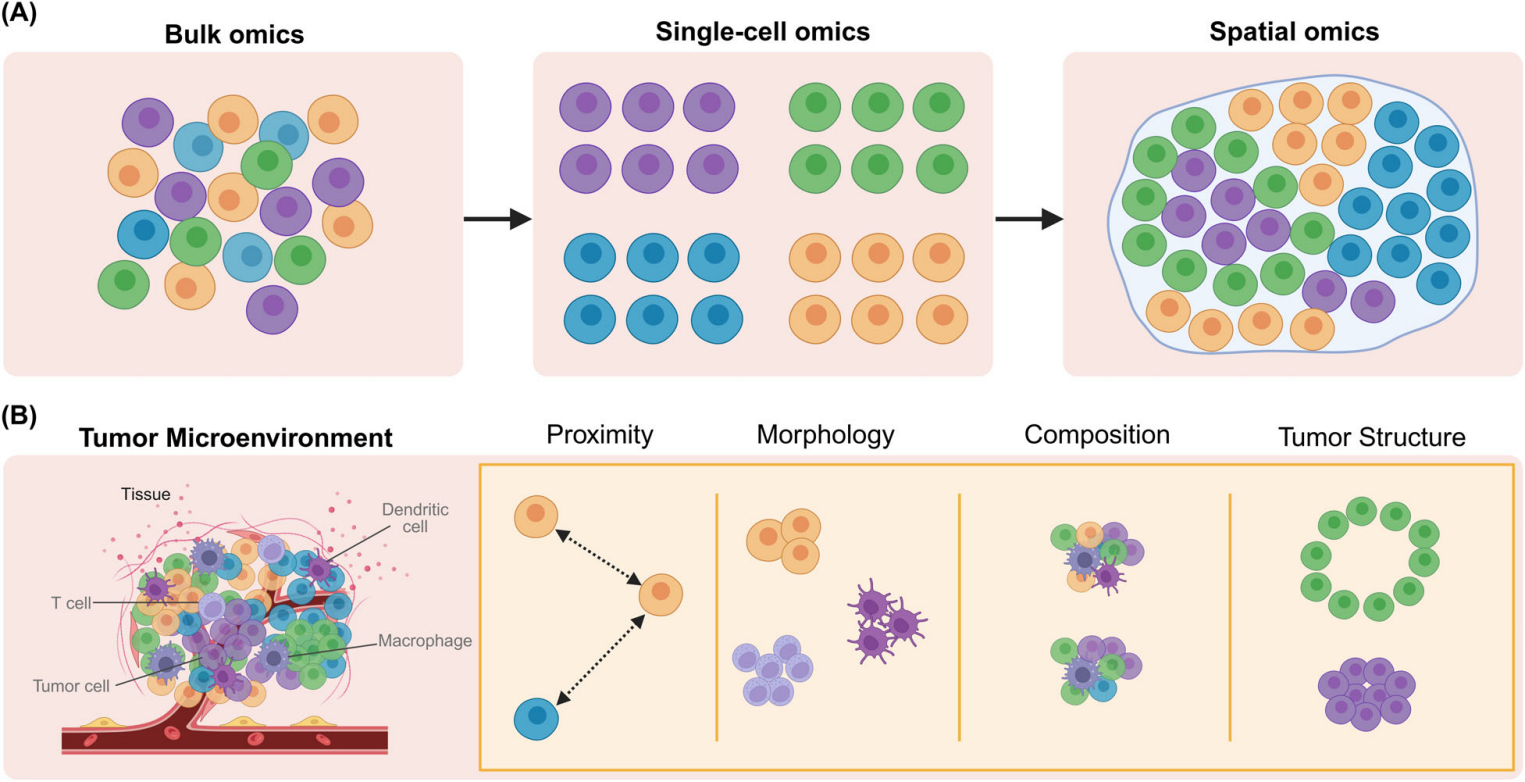
Spatial biology reveals the organization of cancer pathways within intact tissues. (A) Comparison of bulk, single‐cell, and spatial omics approaches. Bulk and dissociated single‐cell methods capture molecular profiles but lose tissue architecture, whereas spatial omics preserves spatial context, enabling molecular measurements to be mapped within intact tumor tissue. (B) Key features of the tumor microenvironment that can be resolved by spatial biology, including cell–cell proximity, cellular morphology, tissue composition, and higher‐order tumor structure. Together, these spatial dimensions allow transcriptional and epigenetic states to be linked to defined tumor niches, providing a more comprehensive understanding of cancer pathways. Created in BioRender. Crespo García, E. (2026) https://BioRender.com/sp4xjj2 [Correction added on 05 August 2026 after first online publication: The BioRender license has been updated.]

Traditional genomic and epigenomic approaches such as bulk sequencing and dissociated single‐cell assays have provided groundbreaking insights into the study of cancer biology. Nevertheless, by altering the tissue architecture, they lose information about where specific regulatory processes occur [39]. This challenge is particularly critical for epigenetics, as chromatin accessibility, histone modifications, and DNA methylation highly respond to microenvironmental signals [40], Fig. 1A, a problem that can now be resolved at single‐cell resolution [41].

Spatial biology addresses this issue by providing key information on cellular proximity (cell–cell interactions and the spatial distribution of cell types within tumors), morphology (the identification and functional characterization of distinct cell phenotypes), composition (the cellular components of the tumor microenvironment and their therapeutic relevance), and tissue structure (the three‐dimensional organization of tumor regions and niches) [42, 43, 44, 45, 46]. In cancer, spatial resolution allows the direct linking of transcriptional [47] and epigenetic [40] states to defined tumor niches, like invasive fronts, hypoxic cores, immune‐excluded regions, and perivascular areas, Fig. 1B. In consequence, a proper understanding of cancer pathways requires not only the identification of molecular alterations but also the mapping of their spatial organization within tumors [48].

## 2 Spatial transcriptomics in oncology: Gene expression as a surrogate for epigenetic states

**Fig. 2:**
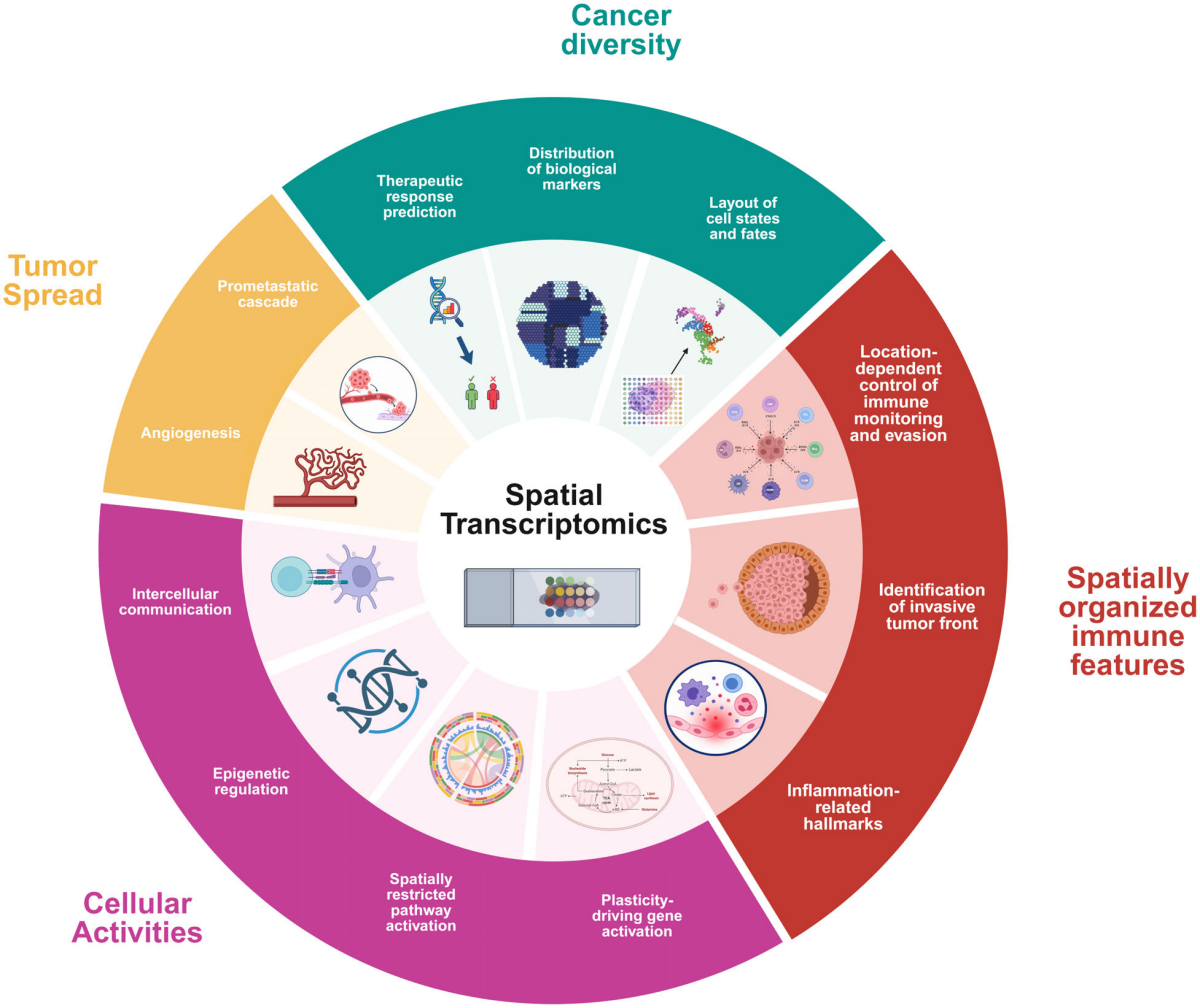
Biological insights enabled by spatial transcriptomics in cancer. Circular schematic summarizing the major biological domains that can be resolved by spatial transcriptomics. Spatially resolved gene expression enables the characterization of cellular responses, tumor heterogeneity, and spatially organized immune programs within intact tissues. By preserving tissue architecture, spatial transcriptomics links transcriptional states to tumor niches and microenvironmental interactions, providing insights into cancer organization, progression, and therapeutic response that are inaccessible to bulk or dissociated single‐cell approaches. Created in BioRender. Crespo García, E. (2026) https://BioRender.com/7yxc1g5 [Correction added on 05 August 2026 after first online publication: Both the figure and the BioRender license have been updated.]

Spatial transcriptomics (ST) technologies facilitate the quantification of messenger RNA (mRNA) transcripts inside intact tissue sections, providing a spatially resolved view of gene expression in cancer and its surrounding microenvironment [49]. By preserving tissue architecture, ST allows the identification of spatially defined cellular programs, tumor niches, and microenvironmental interactions that are invisible to bulk or dissociated single‐cell methodologies. The usage of ST across multiple tumor types has demonstrated considerable potential for precision oncology, including tumor classification [50], identification of invasive fronts [51], characterization of immune–tumor interactions [52], and prediction of therapeutic responses [53], Fig. 2. A diverse ecosystem of ST platforms is currently available, including Xenium and Visium (10× Genomics), Stereo‐seq (STOmics), CosMx SMI and GeoMx DSP (NanoString) [54, 55], as well as imaging‐based methods such as seqFISH and MERFISH (Vizgen) [54].

Even though transcriptomics does not directly measure epigenetic modifications, gene expression patterns often reflect underlying chromatin accessibility, enhancer activity, and transcription factor binding [56]. In this context, transcriptomic overviews provide an indirect readout of regulatory activity and cellular state. Moreover, probing epigenomic–transcriptomic relationships at the single‐cell level have revealed crucial regulators of stemness, cellular plasticity, and state transitions in both normal and malignant tissues. As a result, spatial transcriptomics has frequently been employed as a proxy for epigenetic states in cancer, enabling inference of regulatory programs across spatially defined tumor regions [48, 57]. However, such presumptions remain indirect and cannot fully visualize the regulatory mechanisms that establish, constrain, or stabilize transcriptional outputs.

Although our understanding of gene expression landscapes in cancer has been transformed by spatial transcriptomics, transcription represents only the downstream outcome of a multilayered regulatory hierarchy [58]. Epigenetic mechanisms including chromatin accessibility, histone modifications, and DNA methylation define transcriptional potential, encode regulatory memory, and intermediate cellular responses to microenvironmental cues such as hypoxia, inflammation, and mechanical stress [40]. It is important to note that while transcriptional states may be transient, epigenetic landscapes often persist after cell division, consequently making up the cornerstone of tumor evolution, lineage commitment, and therapeutic resistance [40, 59]. These limitations have driven the development of spatially resolved epigenomic technologies that directly interrogate chromatin states in intact tissues. By enabling the spatial mapping of regulatory programs at the level of chromatin organization, these approaches generate a mechanistic framework to understand how tumor niches impose and maintain epigenetic states that drive cancer progression [60, 61, 62].

Before the arrival of specific spatial epigenomic technologies, researchers tried to reconstruct spatial regulatory landscapes by integrating bulk epigenomic and transcriptomic datasets with tissue‐resolved analyses [63, 64]. One of the first approaches combined bulk microarray‐based genomic and gene expression profiling with public epigenomic resources such as ENCODE, which allowed the association of expression quantitative trait loci (eQTLs) with putative open chromatin regions and regulatory elements [64]. In order to preserve some degree of spatial context, laser microdissection (LMD) was frequently used to isolate anatomically distinct tumor regions (including invasive fronts, tumor cores, and epithelial surfaces) preceding bulk molecular profiling and enabling what can be considered a “pseudo‐spatial” epigenomic analysis [65].

Other studies projected findings from bulk ATAC‐seq datasets back onto tissue architecture using complementary spatial validation techniques. For example, differentially accessible regions (DARs) associated with genes such as EGR1 were validated through approaches including RNAscope and *in situ* hybridization (ISH), providing spatial insight into regulatory patterns identified from bulk analyses [64]. Aligned to this, computational methods based on gene activity scores, which integrate ATAC‐seq signals across promoter and gene body regions, generated transcriptome‐like regulatory profiles that could be aligned with early spatial transcriptomic maps [66].

Although indirect, these initial studies laid important groundwork for the field of spatial epigenomics. By attempting to place chromatin‐associated regulatory programs back into their native tissue context, they provided key conceptual and methodological basic foundations for the development of modern *in situ* epigenetic profiling technologies.

## 3 Spatial epigenomics: Direct mapping of chromatin and DNA modifications in tumors

Spatial epigenomics comprises approaches that interrogate chromatin organization and DNA‐associated modifications that regulate gene activity without changing the underlying DNA sequence [67]. At a molecular level, genomic DNA is packaged into nucleosomes (the fundamental units of chromatin) which are then organized into higher‐order structures that dynamically regulate DNA accessibility. An assorted array of epigenetic modifications, including histone acetylation and methylation, DNA methylation, and histone ubiquitination, play a central role in the building of chromatin architecture and control of regulatory element activity [68, 69]. These modifications influence essential cellular processes such as DNA replication, transcription, repair, and recombination. Dysregulation of these modifications is a hallmark of cancer development and progression [67]. In contrast to other omics layers, the interpretation of epigenomic data relies heavily upon computational and bioinformatic frameworks to deduce regulatory mechanisms from complex, high‐dimensional datasets. The development of robust, scalable, and reproducible experimental and analytical workflows is therefore critical to make the integration of epigenomic information with other molecular layers possible. Ultimately, the aim of spatial epigenomics is to construct a comprehensive spatial regulatory landscape by integrating chromatin accessibility, chromatin dynamics, DNA methylation, and gene expression within intact tissues [70].

**Fig. 3:**
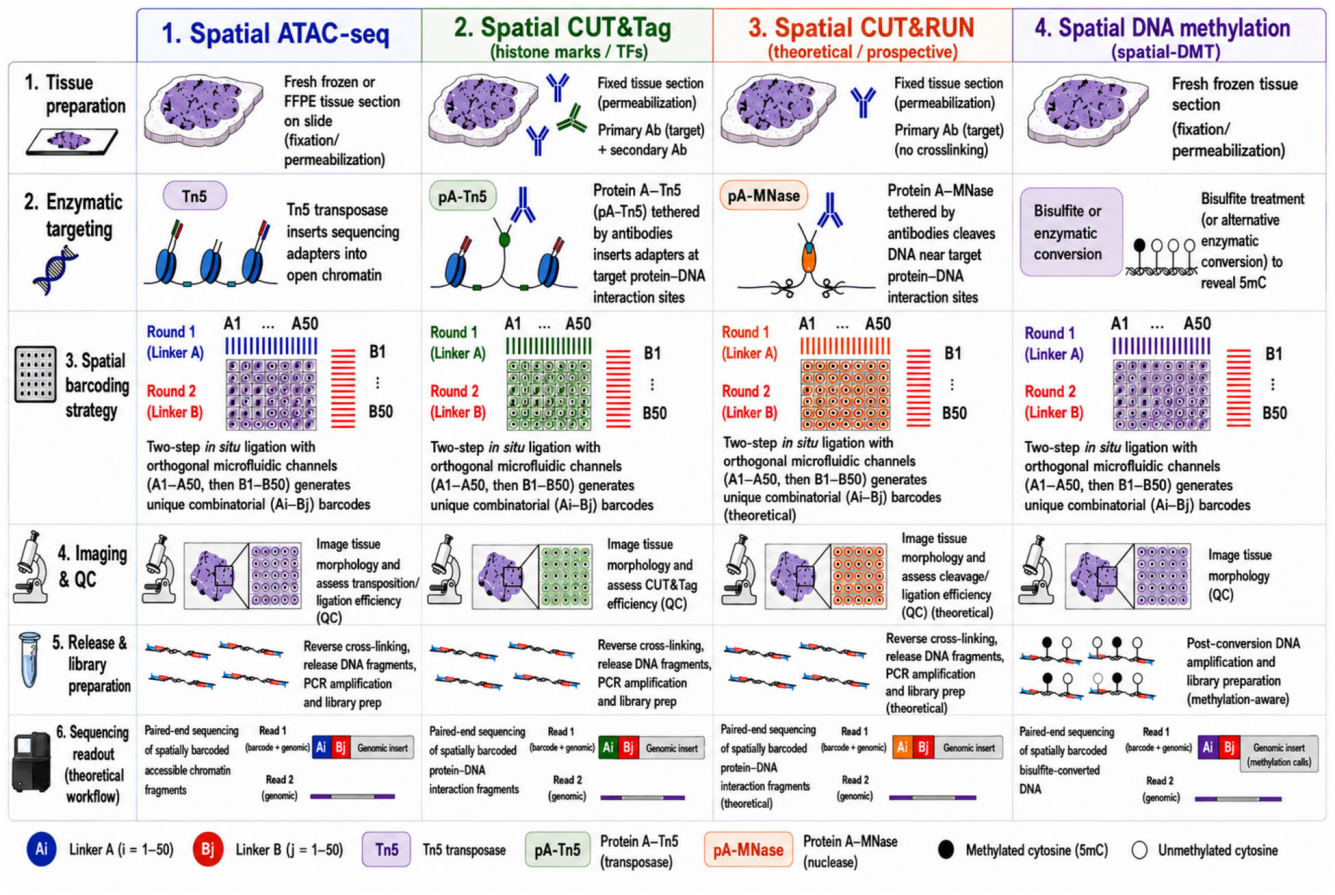
Overview of spatial epigenomic technologies and experimental workflows. Spatial epigenomic approaches enable the mapping of chromatin features and DNA modifications within intact tissue sections while preserving spatial information. The figure compares the experimental workflows of four major spatial epigenomic strategies: spatial ATAC‐seq, spatial CUT&Tag, spatial CUT&RUN (prospective), and spatial DNA methylation profiling (spatial‐DMT). For each method, the workflow is illustrated from tissue preparation and enzymatic targeting through spatial barcoding, imaging and quality control, library preparation, and sequencing readout. Spatial ATAC‐seq uses Tn5 transposase to profile chromatin accessibility, whereas spatial CUT&Tag employs antibody‐guided pA–Tn5 transposition to map histone modifications and chromatin‐associated proteins. Spatial CUT&RUN is shown as a theoretical spatial implementation based on antibody‐tethered pA–MNase cleavage of protein–DNA interaction sites. Spatial DNA methylation profiling combines spatial barcoding with methylation‐sensitive DNA conversion to identify methylated cytosines *in situ*. The spatial barcoding strategy depicted is based on two‐step *in situ* ligation using orthogonal microfluidic channels that generate unique combinatorial spatial barcodes across the tissue section. Created in BioRender. Crespo García, E. (2026) https://BioRender.com/vg2sbb3 [Correction added on 05 August 2026 after first online publication: The BioRender license has been updated.]

Although spatially resolved epigenomic technologies are still in their early stages, they represent a crucial next frontier in cancer research by enabling the direct mapping of regulatory programs within their native tissue context. In the following sections, we review the current landscape of spatial epigenomic methodologies, including spatial ATAC‐seq, spatial CUT&Tag, and emerging spatial CUT&RUN approaches (although this latter technology has not yet been fully implemented), as well as spatial profiling of histone modifications and spatial DNA methylation strategies, Fig. 3 and Table 1.

**Table 1:** Overview of spatial epigenomic technologies in cancer research.

Technology	Molecular layer	Spatial resolution	Scale	FFPE	Sensitivity (low‐abundance targets)	Strengths	Limitations	Cancer applications
**Spatial ATAC‐seq**	Chromatin accessibility	Near single‐cell	High	Yes (spatial FFPE‐ATAC‐seq available)	Moderate	Genome‐wide regulatory mapping; TF motif inference; scalable spatial barcoding	Sparse signal; complex barcoding; indirect functional readout	Breast cancer heterogeneity; glioblastoma spatial niches; pancreatic tumor evolution; CNV‐linked regulatory states
**Spatial CUT&Tag**	Histone modifications; protein–DNA interactions	Single‐cell to near single‐cell	High to moderate	Emerging	High	High signal‐to‐noise; direct histone mark profiling; adaptable to tissues	Microfluidic complexity; tissue variability	Glioblastoma epigenetic niches; lymphoma transformation; breast cancer microenvironment profiling
**Spatial CUT&RUN (emerging)**	Histone modifications; transcription factor binding	Not fully standardized	Moderate	Limited	Very high	High sensitivity; low background	Limited spatial implementation; diffusion of fragments	Not yet widely applied in cancer spatial studies
**Spatial FFPE‐ATAC‐seq**	Chromatin accessibility	Near single‐cell	High	Yes	Moderate	Enables archival clinical samples; preserves spatial architecture	Crosslinking reduces efficiency; sensitivity constraints	Melanoma FFPE tumor heterogeneity; archival tumor profiling
**Spatial DNA methylation (spatial‐DMT)**	DNA methylation + transcriptome	Near single‐cell	Moderate	Emerging	Moderate	Captures stable epigenetic memory; links methylation to transcription	Early‐stage; limited cancer datasets	Mouse developmental systems; emerging tumor epigenetic studies
**Spatial proteomics (PhenoCycler)**	Protein expression (multiplex imaging)	Single‐cell / subcellular	High	Yes	High	Direct cell‐state validation; highly multiplexed protein mapping	Antibody dependency; lower molecular depth vs sequencing	Glioblastoma niche validation (OLIG2, Vimentin, Ki67); tumor microenvironment mapping
**Spatial CITE‐seq**	Protein + transcriptome	Single‐cell	Moderate	Limited	High	Direct multi‐modal profiling; links protein and transcription	Often dissociation‐based; reduced spatial fidelity	Immune–tumor interaction mapping; cell‐state validation

Together, these technologies facilitate the direct spatial interrogation of multiple layers of epigenomic regulation, providing complementary insights into chromatin accessibility, protein–DNA interactions, and DNA methylation landscapes.

### 3.1 Spatial ATAC‐seq: Chromatin accessibility in tissue context

To overcome the limitations of bulk and dissociated single‐cell sequencing approaches, spatial transcriptomics has been developed. It serves as a transformative framework for defining cellular states and functions within their native tissue environment [71, 72, 73, 74, 75]. Clarifying the spatial organization of distinct cell types and functional programs, however, requires not only gene expression profiling but also a direct interrogation of the underlying epigenetic landscape. Chromatin accessibility represents a fundamental regulatory layer, reflecting the activity of promoters, enhancers, and transcription factor binding sites [76]. Therefore, spatial mapping of chromatin accessibility permits the identification of regulatory programs that command tissue organization and cellular function within intact tissue sections.

The assay for transposase‐accessible chromatin using sequencing (ATAC‐seq) was initially developed to profile open chromatin genome‐wide and was afterwards adapted to single‐cell applications [76, 77]. The introduction of ATAC‐seq [78], based on fluorescently labeled Tn5 transposase, provided proof of principle that chromatin accessibility could be interrogated directly within fixed cells and tissues. Together, the combination of regional microdissection with single‐cell ATAC‐seq allowed the profiling of accessible chromatin states from spatially defined tissue compartments [79]. Nevertheless, comprehensive mapping of chromatin accessibility across entire tissue sections with spatial resolution at the cellular level remained technically challenging to implement.

A major breakthrough came with the development of spatial ATAC‐seq [80], in which a spatial barcoding strategy was coupled to Tn5‐mediated insertion of DNA oligomers into accessible genomic loci. This strategy enabled high‐resolution spatial mapping of genome‐wide chromatin accessibility at near single‐cell scale.

In spatial ATAC‐seq, Tn5 transposition is first performed directly on fixed tissue sections, inserting adapters carrying ligation linkers into accessible genomic regions. Spatial barcodes are then added through orthogonally arranged microfluidic channels delivering linker A (A1–A50) and linker B (B1–B50) oligonucleotides in successive rounds of ligation, creating unique combinatorial barcode pairs. The tissue section is later imaged, allowing spatially barcoded accessible chromatin fragments to be associated with tissue morphology. Once a spatially indexed tissue mosaic is formed (of 2500 tiles), reverse crosslinking releases the barcoded DNA fragments, which are then amplified by PCR for library preparation. *In situ* staining of the glass slides permits the evaluation of transposition and ligation efficiency, with imaging serving as a quality control step [80].

Spatial ATAC‐seq combines Tn5 transposase‐mediated insertion of sequencing adapters into accessible genomic regions with spatial barcoding approaches, enabling genome‐wide chromatin accessibility profiling *in situ* and at near single‐cell resolution [80] (Fig. 3). These methodological advances have caused the rapid development and adoption of spatial ATAC‐seq approaches, attaching a regulatory dimension to spatial biology and facilitating the study of chromatin organization in both normal development and disease contexts [64, 81, 82].

An important recent improvement of spatial ATAC‐seq is its adjustment to formalin‐fixed paraffin‐embedded (FFPE) tissues, which results in a spatial FFPE‐ATAC‐seq. FFPE samples represent the gold standard for clinical tissue preservation and constitute an indispensable advantage for retrospective and translational studies. However, broad formalin‐induced crosslinking has historically limited chromatin accessibility profiling in these kinds of samples. This can be fixed by introducing minimal modifications to established spatial ATAC‐seq workflows; this way, spatial FFPE‐ATAC‐seq overcomes these technical obstacles while preserving tissue architecture and spatial information, enabling *in situ* profiling of accessible chromatin in previous clinical specimens [83]. Contrary to earlier FFPE‐compatible chromatin assays which lacked spatial resolution, this tactic makes spatially resolved interrogation of promoter and enhancer accessibility within intact FFPE sections possible. It extends the application of spatial epigenomic analyses to routine pathological material as a result and unlocks an extensive collection of stored human tissues for use in cancer epigenomic analyses.

By producing spatially resolved, genome‐wide maps of accessible chromatin, spatial ATAC‐seq makes possible the identification of active cis‐regulatory elements associated with transcriptionally active genes. Moreover, enrichment analysis of transcription factor (TF) binding motifs within accessible regions allows the inference of regulatory programs operating across defined spatial niches within tumors, offering insight into how microenvironments shape transcriptional control [81].

The latest applications of spatial ATAC‐seq in cancer have provided unprecedented insight into how chromatin accessibility programs are organized inside the tumors and contribute to intratumoral heterogeneity, lineage plasticity, and disease progression. In breast cancer, for example, spatial ATAC‐seq uncovered marked heterogeneity by exposing spatially different tumor clusters characterized by unique regulatory modules and chromatin accessibility states [84]. In HER2‐positive tumors, integrating spatial chromatin accessibility with tissue architecture not only identified HER2‐enriched regions but also highlighted distinct non‐coding regulatory landscapes associated with components of the tumor microenvironment, including myeloid‐related chromatin programs [85]. Moreover, the ability of spatial ATAC‐seq to infer copy‐number variation landscapes showed that spatially separated tumor subclones can arise from a common ancestor while acquiring divergent regulatory dependencies, such as MAPK‐driven or estrogen‐responsive states [84].

Similar insights have emerged in glioblastoma, where spatial epigenomic profiling revealed nested chromatin accessibility niches composed of OPC‐like SOX10‐positive inner regions surrounded by mesenchymal VEGFA‐positive outer regions. These spatially organized niches displayed distinct transcription factor networks and signaling programs, including PDGFB and BMP7, which appear to contribute both to the maintenance of local cellular identities and to communication with neighboring tumor cells. The same study further demonstrated the power of spatial ATAC‐seq to track localized subclonal evolution, uncovering spatially restricted amplifications of oncogenic drivers such as EGFR, MDM2, and CDK4 within glioblastoma cores [81].

In pancreatic neuroendocrine tumors, the integration of single‐nucleus and spatial ATAC‐seq enabled the reconstruction of epigenetic trajectories associated with tumor progression, linking proliferative MYC‐ and FOX‐enriched regions to invasive niches characterized by Snail‐family transcription factors and active KRAS signaling [86]. More recently, the development of spatial FFPE‐ATAC‐seq and epi‐Patho‐DBiT has expanded chromatin accessibility profiling to archived clinical samples, greatly increasing the translational potential of spatial epigenomics [83, 87]. In human melanoma FFPE specimens, these approaches resolved both tumor and stromal heterogeneity and identified spatially distinct accessibility patterns at clinically relevant loci, including BRAF, KIT, and NRAS [83].

Collectively, these studies highlight the unique ability of spatial ATAC‐seq to connect chromatin accessibility landscapes with tissue architecture, tumor evolution, and microenvironmental interactions, providing a more comprehensive view of cancer biology than is possible with dissociated‐cell approaches alone.

The growing complexity of spatial epigenomic datasets has also stimulated the development of specialized computational tools designed to extract biologically meaningful information from highly sparse chromatin accessibility data. Among these, graph‐based methods such as Descart integrate epigenomic signals with spatial coordinates to identify chromatin accessibility peaks that vary across tissue regions. By explicitly accounting for data sparsity, these approaches can uncover spatial peak modules and gene–peak interactions linked to tumor microenvironmental organization and regulatory heterogeneity. As a result, they facilitate the reconstruction of spatial regulatory networks and enhance our ability to interpret the chromatin accessibility programs that shape cancer tissue [88].

Both academic and commercial platforms, including recent developments by AtlasXomics [89], are accelerating the implementation of spatial ATAC‐seq in cancer research and expanding its applicability to translational oncology.

### 3.2 Spatial CUT&tag and CUT&RUN: Mapping protein–DNA interactions

A major goal of spatial epigenomics is to characterize protein–DNA interactions and chromatin states directly within intact tissues. To achieve this, several spatial approaches have adapted already‐established chromatin profiling methods. Most notable among these is CUT&Tag and, possibly in the future, CUT&RUN, for use in tissue sections while preserving spatial information [60, 90, 91]. Both techniques rely on targeting of specific chromatin features through the use of antibody‐guiding. They differ, however, in how genomic DNA is processed once the target has been recognized.

CUT&RUN (Cleavage Under Targets and Release Using Nuclease) uses a Protein A–micrococcal nuclease (MNase) fusion protein to cleave DNA selectively adjacent to antibody‐bound chromatin regions. Because the method is performed in permeabilized cells or nuclei without crosslinking or extensive chromatin fragmentation, background signal is substantially reduced compared with conventional ChIP‐based approaches. The result of this is highly specific chromatin maps [92, 93]. As part of the broader family of chromatin profiling technologies that includes ChIP‐seq, ATAC‐seq, and FAIRE‐seq, CUT&RUN can be used to interrogate histone modifications, transcription factors, and chromatin‐associated cofactors depending on the antibody employed [92].

In a typical experiment, permeabilized cells or nuclei are immobilized on magnetic beads and incubated with an antibody directed against the chromatin feature of interest. A Protein A–MNase fusion protein is then recruited to the antibody–target complex. Following calcium activation, MNase cleaves the DNA flanking the bound protein and releases short fragments corresponding to protein–DNA interaction sites. These fragments are subsequently purified and used for sequencing library preparation. Owing to its low background signal, CUT&RUN generally requires fewer sequencing reads than ChIP‐seq while maintaining high sensitivity [92].

Spatial CUT&Tag takes a different approach. Instead of releasing DNA fragments, it uses an antibody‐tethered Protein A–Tn5 transposase to insert sequencing adapters directly at target loci *in situ*, eliminating the need for DNA recovery and diffusion steps [60, 94], Fig. 3. This modification has important practical advantages, including improved signal‐to‐noise ratios, higher spatial resolution, and greater scalability. As a result, CUT&Tag has become the predominant strategy for spatial profiling of histone modifications, particularly in complex tissues such as tumors [60, 90, 95].

Before the introduction of CUT&Tag‐based spatial methods, most spatial chromatin assays were largely restricted to Tn5‐based accessibility profiling approaches such as ATAC‐seq. The first spatial CUT&Tag study, reported in 2022, combined *in situ* CUT&Tag chemistry with droplet‐based single‐cell technologies capable of recording cellular position within tissues [60]. Subsequent studies incorporated deterministic barcoding strategies to improve spatial resolution and throughput, establishing a framework that is now widely used for profiling histone modifications including H3K27me3, H3K27ac, and H3K4me3 [75, 94, 96, 97].

The typical workflow begins with incubation of a fixed tissue section with a primary antibody targeting the histone modification of interest, followed by a secondary antibody to enhance tethering of the pA‐Tn5 transposome. Upon activation, the transposome inserts sequencing adapters containing ligation linkers at antibody‐bound genomic loci. A first set of DNA barcodes (Ai, i = 1–50) is delivered across the tissue through microchannel‐guided flow, enabling *in situ* ligation to the adapters. Subsequently, a second set of barcodes (Bj, j = 1–50) is applied via perpendicular microchannels, generating a two‐dimensional grid of tissue “pixels,” each defined by a unique Ai–Bj barcode combination. After imaging to correlate morphology with the spatial epigenomic map, DNA fragments are recovered through cross‐link reversal to complete library preparation [60]. This workflow preserves native tissue architecture while enabling high‐resolution spatial mapping of chromatin states [98].

Despite these advances, spatial CUT&Tag remains technically demanding. The structural complexity and heterogeneity of tissue sections can lead to barcode leakage, microchannel blockage, or uneven barcode distribution. Reproducibility and spatial fidelity are compromised as a result. This is particularly the case when there exist cavities or uneven surfaces. Moreover, reliance on microfluidic devices increases technical complexity and limits scalability.

To overcome these constraints, a next‐generation strategy termed Super‐CUT&Tag has recently been introduced. This method replaces microfluidic barcoding with a solid‐phase, ultra‐dense DNA barcode array combined with three‐dimensional PAMAM dendrimers to enhance chromatin DNA capture efficiency. The use of pre‐fabricated barcoded slides allows quality control prior to tissue placement, significantly reducing experimental failure and improving reproducibility. Compared with conventional spatial CUT&Tag, Super‐CUT&Tag achieves a several‐fold increase in sensitivity for H3K27ac profiling. Application of this platform to mouse embryos (E10.5–E14.5) enabled identification of region‐specific enhancers demarcating developmental compartments and, at 15 μm resolution, resolved heterogeneity of H3K27ac across distinct layers of the developing forebrain cortex. Integration of spatial epigenomic data with matched transcriptomes further revealed the spatiotemporal regulatory circuitry through which Neurod2 governs pallial neuronal identity [99].

Since their introduction, spatial CUT&Tag and its enhanced variants have been primarily applied to developmental systems, where spatially resolved epigenomic regulation is central to lineage specification and tissue patterning. The first spatial CUT&Tag study [60] demonstrated *in situ* profiling of histone modifications in mouse embryos, establishing proof of principle that chromatin states such as H3K27me3 and H3K4me3 could be mapped with spatial resolution across intact tissue sections. Subsequent implementations integrating deterministic barcoding approaches further extended this strategy to embryonic and brain tissues, enabling spatial reconstruction of epigenetic landscapes during neurodevelopment and organogenesis [95, 99, 100].

To date, applications in human cancer specimens remain largely unexplored. Although spatial CUT&Tag holds strong conceptual potential for dissecting epigenetic heterogeneity within tumor niches and tumor–immune microenvironments, most published studies have focused on developmental or model tissues rather than pathological oncology samples. Consequently, the systematic implementation of spatial CUT&Tag in cancer biology remains an emerging area of investigation.

Although only a limited number of cancer‐focused studies have been reported to date, early applications of spatial CUT&Tag have already highlighted the value of spatial chromatin profiling in oncology. Using the epi‐Patho‐DBiT platform, spatial FFPE‐CUT&Tag was applied to archived human lymphoma specimens to investigate the transformation of follicular lymphoma into diffuse large B‐cell lymphoma (DLBCL) [87]. This approach uncovered extensive epigenetic remodeling during disease progression, including altered spatial distributions of the active histone mark H3K4me3 and the repressive mark H3K27me3 at genomic regions linked to malignant transformation [87]. In particular, transformed DLBCL regions displayed increased H3K27me3 occupancy at amplified chromosome 2 loci harboring tumor‐promoting genes. This pattern coincided with transcriptional dysregulation and disease progression [87].

These findings demonstrate how spatial CUT&Tag can connect regional chromatin states with the molecular processes driving tumor evolution. In doing so, it provides spatial context that is often lost in conventional epigenomic analyses. At the same time, emerging multimodal technologies such as SPACE‐seq point to future opportunities for expanding spatial histone profiling. Because SPACE‐seq incorporates polyA‐loaded transposome strategies that were originally developed for spatial ATAC‐seq, similar concepts could potentially be adapted to CUT&Tag‐based workflows. This could extend spatial histone modification mapping to a broader range of cancer tissues and tumor microenvironmental niches [101].

As the technology continues to mature, spatial CUT&Tag is likely to become an increasingly valuable tool for studying how histone modification landscapes are organized across tumors and how they contribute to cellular plasticity, immune regulation, and therapeutic resistance.

While spatial CUT&Tag has become the dominant strategy for profiling protein–DNA interactions in intact tissues, the direct adaptation of CUT&RUN for spatial applications remains largely experimental. To date, no studies have fully implemented spatial CUT&RUN in tissue sections, and methodological development is ongoing to address challenges such as diffusion of cleaved DNA fragments and the requirement for high‐resolution barcoding [45, 46]. Nevertheless, spatial CUT&RUN holds promise as a complementary approach to spatial CUT&Tag, potentially enabling high sensitivity mapping of low‐abundance transcription factors and proteins associated with chromatin. It is expected that the spatial epigenomics toolkit will expand and our understanding of chromatin regulation within native tissue contexts will deepen as innovation continues in this area.

### 3.3 Spatial DNA methylation

Being one of the most widely studied epigenetic mechanisms that regulates cell identity and gene expression, DNA methylation holds a central role in lineage specification and cellular differentiation by influencing chromatin structure and the accessibility of regulatory regions to transcriptional machinery [43, 102]. DNA methylation patterns are dependent on context to a high degree and vary greatly across cell types, developmental stages, and environmental conditions [103]. Cancer [40, 104], inflammatory diseases [105] and autoimmune disorders [106] can all be attributed, at least in part, to the disruption of methylation patterns. With age, changes in DNA methylation also accumulate and have been used as biomarkers of biological aging through the development of epigenetic clock models [107, 108].

The rapid expansion of spatial multi‐omics technologies has transformed our ability to study molecular processes within intact tissues. Spatially resolved measurements of gene expression, chromatin accessibility, histone modifications, and protein abundance have proven to be particularly useful in understanding tissue organization and cellular interactions in the context of their native microenvironment [75, 90, 109]. Yet, despite the fundamental role it plays in gene regulation, DNA methylation notably remained absent from the spatial epigenomics toolbox. As a result of this absence, little was known about the organization of methylation landscapes across tissues or how they relate to local transcriptional and chromatin states.

This situation has begun to change with the development of approaches capable of measuring DNA methylation in a spatially resolved manner, Fig. 3. Among the first of these is spatial‐DMT, which enables the joint profiling of the DNA methylome and transcriptome within the same tissue section. Due to its ability to simultaneously capture these two molecular layers, the method offers a unique opportunity to investigate how DNA methylation and gene expression are coordinated across space and time. Through initial applications, complex spatiotemporal relationships between methylation and transcriptional activity were revealed. This highlighted the potential of spatial methylome profiling to uncover hierarchical regulatory programs operating within intact tissues [110].

At present, spatial DNA methylation studies remain largely confined to mouse embryos and postnatal brain tissues, and high‐resolution applications in human cancer specimens have yet to become routine [110]. Nevertheless, the relevance of this technology to the field of oncology is evident. Aberrant DNA methylation is a defining feature of many cancers, contributing to tumor heterogeneity, lineage plasticity, and large‐scale chromatin reorganization. Hypermethylation‐mediated silencing of tumor suppressor genes such as PTEN represents just one example of how altered methylation landscapes can stabilize malignant cellular phenotypes and promote disease progression.

Interest in incorporating DNA methylation into spatial multi‐omic analyses is therefore growing. Methylation information has begun to be used alongside chromatin accessibility and transcriptional data to better understand spatial regulatory transitions across tissues and tumor regions in recent studies [86, 111]. In parallel to these developments, emerging integrated platforms such as SPACE‐seq have been created with adequate flexibility to accommodate future DNA methylation measurements together with transcriptomic and chromatin accessibility profiling [101]. These technologies are expected to provide a more comprehensive view of epigenetic regulation *in situ* as they mature and are refined. The potential uses of this include helping to unravel how different epigenetic layers interact to shape tumor evolution, immune escape, metastatic dissemination, and therapeutic resistance [101, 111].

### 3.4 Spatial proteomics and multimodal integration in spatial epigenomics

In order to be complete, an understanding of tumor biology must include integration of epigenomic information with protein‐level phenotypes that define functional cellular states. Spatial proteomics thus represents a key complementary layer to spatial epigenomics, reflected in its ability to enable validation of chromatin and transcription‐based inferences within intact tissues.

Multiplex protein imaging approaches such as PhenoCycler enable simultaneous detection of multiple protein markers at single‐cell resolution within the same tissue section. In glioblastoma (GBM), this technology has been used to validate spatially distinct tumor cell states, including OPC‐like and mesenchymal‐like populations marked by OLIG2 and Vimentin, respectively. These analyses confirmed that such niches are spatially organized across both tumor core and margin 8, with proliferation markers such as Ki67 further identifying OPC‐like and intermediate states as key proliferative compartments [81].

In parallel, spatial CITE‐seq enables joint profiling of protein abundance and transcriptomes at cellular resolution, providing a direct link between surface phenotypes and transcriptional programs [88]. This is particularly useful for refining cell‐state definitions derived from epigenomic or transcriptomic data.

Protein measurements are also increasingly incorporated into spatial epigenomic workflows. Standard spatial ATAC‐seq protocols include immunostaining steps prior to chromatin profiling, allowing protein signals to be spatially registered alongside chromatin accessibility within the same tissue section [85]. This integration improves cell type annotation and enhances interpretation of regulatory landscapes.

In breast cancer, such multimodal strategies have been used to associate HER2‐enriched chromatin accessibility states with protein‐level tumor architecture and immune infiltration, including the presence of myeloid cells in the tumor microenvironment [85]. These findings highlight the value of linking regulatory states to functional protein outputs in spatial contexts.

Future multimodal frameworks, including extensions of SPACE‐seq, are expected to incorporate spatial protein measurements alongside chromatin accessibility and gene expression [101]. More broadly, emerging spatial analyses increasingly rely on integrated datasets combining epigenomic, transcriptomic, and proteomic information from the same cells to improve resolution of tumor heterogeneity and regulatory interpretation [84].

Overall, spatial proteomics provides a critical bridge between epigenomic regulation and functional phenotype, enabling more accurate biological interpretation of spatial multi‐omic landscapes in cancer.

## 4 Challenges and future perspectives in spatial epigenomics

The field of spatial epigenomics remains in its early stages despite the rapid conceptual and technological progress that has been made. This is especially the case in the context of cancer. Only a limited number of studies have applied spatial epigenetic profiling so far. The ones that have done so were mainly spatial ATAC‐seq, spatial CUT&Tag, and spatial DNA methylation. Additionally, most of these have focused on developmental systems or non‐malignant tissues. This means that when it comes to cancer, our view of how spatially organized epigenetic programs contribute to tumor evolution, heterogeneity, and treatment response remains pretty fragmented at best. In practice, this gap becomes even more pronounced given the extreme heterogeneity of tumors, where genetically and epigenetically distinct subclones coexist in tightly confined spatial niches, producing regulatory landscapes that are difficult to resolve with current technologies.

Broader adoption is slowed down by a number of technical limitations. Sensitivity remains an issue, especially when profiling low‐abundance chromatin‐associated factors. In addition, there often tends to be a trade‐off between spatial resolution and genomic coverage that is unavoidable. Many workflows require specialized equipment and substantial technical expertise, which limits accessibility because of how experimentally demanding they are. On top of this, compatibility with certain clinical samples or downstream assays can be restricted as most methods rely on fixed tissue sections. In a translational setting, these challenges are further complicated by tumor composition itself: clinical specimens rarely consist of pure cancer cell populations and instead include immune cells, fibroblasts, endothelial cells, and other stromal components, all of which blur the assignment of spatial epigenomic signals to specific cell types. Improving reproducibility, standardizing protocols, and reducing input requirements will therefore be key steps toward wider application in cancer research.

An additional layer of difficulty is introduced when performing computational analysis. Spatial epigenomic datasets are high‐dimensional and sparse. Dedicated analytical frameworks are required to extract meaningful biological structure from the sets. These frameworks must be able to jointly handle chromatin accessibility, histone modifications, DNA methylation, and transcriptional outputs, all within a spatial framework. While progress on this challenge is being made, robust solutions for normalization, spatial domain detection, and multimodal integration are still changing and being developed. Another unresolved issue is the lack of comprehensive reference atlases for spatial epigenomic states in human tissues, particularly in cancer, which makes cross‐study comparison and biological interpretation more challenging. Tumor genomes add yet another complication: widespread copy‐number aberrations can distort chromatin accessibility and histone modification signals simply by altering DNA dosage, creating apparent regulatory variation that is not truly epigenetic in origin and therefore requires careful correction.

In parallel with these challenges, spatial multi‐omics approaches are beginning to change what is technically possible. Several platforms now allow chromatin accessibility or histone modifications to be profiled together with gene expression in the same tissue section, linking regulatory activity directly to transcriptional output in spatial context [90]. Methods such as spatial ATAC–RNA‐seq and spatial CUT&Tag–RNA‐seq use either deterministic barcoding or microfluidic spatial indexing to map epigenomic and transcriptomic layers at near–single‐cell resolution while preserving tissue architecture [100]. More recently, unified chemistries compatible with commercial spatial transcriptomics platforms have further simplified multi‐omic profiling by enabling simultaneous capture of chromatin accessibility, gene expression, and lineage information [101]. Rather than relying on computational alignment of independently generated datasets, these approaches provide direct, genome‐wide measurements of how spatially organized regulatory landscapes shape transcriptional programs *in situ*. In tumors, this level of integration is particularly informative for dissecting functional states within distinct microenvironmental niches, such as hypoxic regions, invasive margins, perivascular zones, and immune‐excluded areas, where epigenetic state is tightly linked to local cellular ecology.

Looking ahead, both the scale and the resolution of spatial epigenomics in cancer will likely expand as a result of continued methodological improvements. It will be possible to interrogate rare but biologically important populations with higher sensitivity, better multiplexing, and improved spatial resolution. This could include cancer stem cells, therapy‐resistant clones, and invasive subclones. At the same time, it will be essential to integrate spatial epigenomics with spatial transcriptomics, proteomics, and imaging to build a more complete systems‐level view of tumor biology. As these technologies mature and move into larger clinical cohorts, they are expected to play an increasingly important role in biomarker discovery and precision oncology by revealing regulatory programs that are invisible in dissociated‐cell approaches. Ultimately, progress in this field will depend on explicitly linking spatial epigenetic states to clonal architecture, immune interactions, and microenvironmental gradients, enabling reconstruction of tumor evolution in space and identification of epigenetically defined resistant niches.

## 5 Conclusions

Spatial epigenomics has rapidly evolved from a conceptual framework into a diverse collection of technologies capable of mapping the regulatory landscape of tissues while preserving their spatial organization. Although many of these approaches are still relatively new, they have already begun to change how we investigate complex biological systems, particularly those in which cellular context is just as important as molecular identity.

In cancer, this is especially relevant. Tumors are highly heterogeneous tissues where malignant cells continuously interact with immune, stromal, and vascular compartments, creating distinct ecological niches that influence disease progression and therapeutic response. Spatial epigenomic technologies provide an opportunity to study these interactions directly, revealing regulatory mechanisms that remain inaccessible using bulk sequencing or dissociated single‐cell approaches. As spatial multi‐omic strategies continue to develop, it will become increasingly feasible to examine chromatin state, DNA methylation, gene expression, and protein activity simultaneously within the same tissue section, providing a far more complete picture of tumor biology.

Despite this progress, several challenges still need to be addressed before these methods become routine research and clinical tools. Improvements in sensitivity, spatial resolution, protocol standardization, and computational analysis will all be necessary to increase reproducibility and facilitate comparisons across studies. Equally important is the development of robust analytical frameworks capable of integrating the multiple molecular layers generated by these technologies into biologically meaningful models.

The pace of innovation in the field suggests that many of these limitations are likely to be overcome in the coming years. As spatial epigenomics becomes more accessible and is applied to larger patient cohorts, it is expected to substantially improve our understanding of tumor evolution, cellular plasticity, and the dynamic interactions that shape the tumor microenvironment. Ultimately, combining spatial epigenomics with complementary molecular and imaging approaches will provide a more comprehensive view of cancer biology and contribute to the development of more precise diagnostic, prognostic, and therapeutic strategies.

## Conflict of interest

ECG declares no conflicts of interest. M. E. reports grants from Incyte and personal fees from Quimatryx, Eucerin, and L'Oreál outside the submitted work.

## Author contributions

ECG and ME wrote, reviewed, and edited the original draft.
